# TAVI Performance at a Single Center over Several Years: Procedural and Clinical Outcomes

**DOI:** 10.3390/medicina62010204

**Published:** 2026-01-18

**Authors:** Huseyin Dursun, Bihter Senturk, Tugce Colluoglu, Cisem Oktay, Hacer Uysal, Husna Tuğçe Simsek, Sercan Karaoglan, Zulkif Tanriverdi, Dayimi Kaya

**Affiliations:** 1 Department of Cardiology, Faculty of Medicine, Dokuz Eylul University, Izmir 35340, Turkey; drhuseyindursun@gmail.com (H.D.); bihter.senturk@deu.edu.tr (B.S.); cisem.oktay@deu.edu.tr (C.O.); haceruuysal@gmail.com (H.U.); husnatugce.simsek@deu.edu.tr (H.T.S.); sercan.karaoglan@deu.edu.tr (S.K.); dayimikaya@gmail.com (D.K.); 2 Department of Cardiology, Faculty of Medicine, Karabuk University, Karabuk 78200, Turkey; 3 Department of Cardiology, Faculty of Medicine, Harran University, Sanliurfa 63300, Turkey; ztverdi@gmail.com

**Keywords:** aortic stenosis, transcatheter aortic valve implantation, outcomes

## Abstract

*Background and Objectives:* Transcatheter aortic valve implantation (TAVI) has become the mainstay of treatment for symptomatic aortic stenosis (AS) in patients over 70 years of age. It is also indicated for younger patients with significant comorbidities, for valve-in-valve interventions, and in selected patients with severe aortic insufficiency. We aimed to evaluate procedural and clinical outcomes of transfemoral TAVI performed over the course of 12 years by the same operators using different bioprosthetic valves. *Materials and Methods:* Between 2012 and 2023, 375 patients underwent TAVI in our clinic, with six types of bioprosthetic valves (Edwards Sapien XT, Medtronic Valves [CoreValve and Evolut R], Portico, Myval, Acurate Neo, and Direct Flow Medical). A transfemoral approach was used in all patients. The procedural and clinical outcomes were defined according to Valve Academic Research Consortium-3 (VARC-3) criteria. *Results:* The mean age of the patients was 78.4 ± 7.3, and their median STS score was 4.2 (2.9–5.9). Of the 375 patients, 361 had severe AS, 4 had severe aortic insufficiency, 5 were valve-in-valve, and 5 were valve-in-TAVI. Seven patients required a second valve implantation: four due to embolization of the prosthetic valve and three due to deep implantation of the prosthetic valve. Based on the VARC-3 criteria, the rates of technical success and device success were 90.4% and 85.3%, respectively. Major vascular complications were observed in 18 (4.8%) patients. Also, 42 (11.2%) patients required permanent pacemaker implantation. The incidence of moderate or worse paravalvular leak was 2.9%. The peri-procedural, 30-day, 1-year, and 5-year mortality rates were 5.1%, 4.3%, 15.2%, and 45.6%, respectively. STS scores (HR:1.129, 95%CI: 1.068–1.192, *p* < 0.001) and post-TAVI acute kidney injury (HR:3.993, 95%CI:1.629–9.785, *p* = 0.002) were detected as independent predictors of mortality in Cox regression analysis. *Conclusions:* This registry demonstrated the evolution of TAVI procedures at a single center over 12 years. A high level of collaboration between experienced operators and innovations in devices seem to be the key features for achieving high procedural success and low complication rates.

## 1. Introduction

Transcatheter aortic valve implantation (TAVI) has become a well-established therapeutic option for severe aortic stenosis (AS) patients over 70 years of age with suitable anatomy and for younger inoperable or surgically high-risk patients [1]. Since the first successful implantation by Cribier et al. in 2002, the number of TAVI procedures has rapidly increased worldwide and in our country [2,3,4,5]. Besides AS, the indications for TAVI have expanded over time. The procedure has also been performed in degenerated surgical or TAVI valves (valve-in-valve) and in cases of severe aortic insufficiency in limited numbers of patients who are inoperable and have suitable anatomy [6,7,8]. Many bioprosthetic valves have been developed so far, beginning with first-generation devices such as the balloon-expandable Edwards SAPIEN XT valve (ESV; Edwards Lifesciences, Irvine, CA, USA) and the self-expanding Medtronic CoreValve (MCV, Medtronic Inc., Minneapolis, MN, USA). The results of the early TAVI experience revealed the necessity of advances in bioprosthetic valves and delivery system designs, along with a minimalist approach for TAVI to provide procedural advantages and lower complication rates [4,9,10].

The coordination and experience of the heart team are undoubtedly among the most important factors in a successful TAVI operation [11]. TAVI procedures were first performed in Turkey by 2009, and they have been successfully employed in our department since 2012 [3]. We began with MCV implantation, and our ESV implantation rates then increased steadily. The next-generation Medtronic Evolut R and other devices such as the Direct Flow Medical valve (DFM, Direct Flow Medical Inc., Santa Rosa, CA, USA), Portico valve (St Jude Medical, St. Paul, MN, USA), Myval (Meril Life Sciences, Vapi, India), and Acurate Neo valve (Boston Scientific, Marlborough, MA, USA) have since been implanted in different numbers. Our department has performed one of the largest TAVI series in our country using different types of bioprosthetic valves.

We aimed to present our 12-year single-operator experience using various bioprosthetic valves and assess the procedural details and outcomes of TAVI based on the VARC-3 criteria [12].

## 2. Materials and Methods

### 2.1. Study Design and Data Collection

A consecutive series of 375 patients who underwent TAVI between 1 June 2012, and 1 December 2023, was retrospectively included in this study. All patients were evaluated by a multidisciplinary heart team including two cardiologists, two cardiac surgeons, and one cardiac anesthesiologist. The decision to perform TAVI was made according to the current European guidelines [13,14,15]. Surgical risk was calculated via the Society of Thoracic Surgeons predictive risk for mortality (STS PROM) score and the Logistic European System for Cardiac Operative Risk Evaluation Scores (EuroSCORE I and EuroSCORE II). Surgical risk was defined as low, intermediate, and high with STS scores of 4%, 4–8%, and >8%, respectively. Other significant comorbidities, such as previous chest radiation, a prior history of coronary artery bypass grafts, porcelain aorta, liver cirrhosis, and frailty, put the patient at high risk [13,16]. All patients who underwent a TAVI procedure during the study period, independent of whether the valve could be implanted, were included in this study.

Peri-procedural death; 30-day, 1-year, and 5-year all-cause mortality; stroke; vascular complications; acute kidney injury; paravalvular leak (PVL); and permanent pacemaker implantation (PPM) requirements were recorded according to the VARC-3 classification [12]. Vascular and access-site-related complications encompassed any adverse events arising from the entry site (femoral artery or vein), the insertion or removal of the device or its components (needle, wire, dilator, sheath, and catheter), or the device delivery process according to the VARC-3 criteria. Neurological imaging techniques (magnetic resonance imaging or computed tomography) were used to diagnose stroke in patients who developed neurological symptoms after TAVI. Acute kidney injury (AKI) was defined according to the temporal change in creatinine levels from baseline to 48 h post-procedure. The severity of PVL was categorized based on its circumferential extent (sum of the circumferential lengths of each regurgitant jet vena contracta/the circumference of the outer edge of the transcatheter valve) as mild (less than 10%), moderate (between 10% and 30%), or severe (more than 30%). Technical success was evaluated based on the VARC-3 criteria as follows: (1) freedom from mortality; (2) successful access, delivery of the device, and retrieval of the delivery system; (3) correct positioning of a single prosthetic heart valve into the proper anatomical location; and (4) freedom from any further surgery or intervention related to the device or to any major vascular, access-related, or cardiac structural complication. Device success according to the VARC-3 criteria included (1) technical success; (2) freedom from mortality; (3) freedom from any further surgery or intervention related to the device or to any major vascular, access-related, or cardiac structural complication; and (4) the valve performing as intended. Deaths were analyzed based on the time that had elapsed since the index procedure [12] and were verified through hospital records and family contacts. This study was approved by the local institutional ethics committee and performed in accordance with the Declaration of Helsinki.

### 2.2. Pre-Procedural Evaluation

Patients were assessed via transthoracic echocardiogram (TTE) for the diagnosis of severe AS according to the current recommendations, and the following measurements were recorded: left ventricular diameters, left ventricular ejection fraction (LVEF), mean and maximum transaortic gradients, aortic valve area (AVA), aortic annulus, and ascending aorta measurements [17,18]. In the first 77 patients, the aortic annulus evaluation was performed by means of transesophageal echocardiography (TEE). Then, multi-slice computed tomography (MSCT) was adapted into clinical use instead of TEE and provided detailed information about the aortic annulus, valve morphology, calcification distribution, sinus Valsalva diameter, sinotubular junction diameter, coronary take-offs from hinge points, and peripheral artery diameters for the access site [19]. All patients underwent coronary angiography (CAG) to assess their coronary arteries before TAVI. Before the clinical implementation of MSCT, aortography was performed to evaluate the aortic annulus and the distance from the coronary ostium to annulus, and peripheral angiography was performed to assess the appropriateness of peripheral access. In the presence of significant (>%70 in epicardial coronary arteries, >%50 for left main) coronary artery disease, a percutaneous coronary intervention was performed before or during the TAVI procedure according to the operators’ preference.

### 2.3. TAVI Procedure

The transfemoral approach was used in all patients. In the first 104 cases, TAVI was performed under general anesthesia. Surgical cutdown was utilized in 94 of these cases; in the remaining 10 cases, the Prostar^®^ XL (ProStar™ XL, Abbott Vascular, Santa Clara, CA, USA) percutaneous closure system was used. Then, we transitioned to a minimalist approach with local anesthesia and deep sedation. In most of the remaining cases, the Perclose Proglide^TM^ (Abbott Vascular) percutaneous closure system was used. A temporary non-balloon-tipped pacemaker was placed in the right ventricular apex, and, if needed, a balloon valvuloplasty was performed under rapid ventricular pacing. The bioprostheses used were the MCV, ESV, Medtronic Evolut R, DFM, Portico valve, Myval, and Acurate Neo valve.

### 2.4. High Implantation Technique

The different design of the MCV usually causes the bioprosthesis to extend a greater degree into the left ventricular outflow tract. Deep implantation may cause sub-annular placement of the bioprosthesis skirt, which decreases annular contact and alters the location of the uncovered parts of the nitinol stent at the level of the annulus. It also exerts greater mechanical stress on the left bundle branch, thus increasing the incidence of PPM. To decrease the frequency of PVL and PPM with the MCV, we therefore attempted to routinely perform a high implantation technique in the standard co-planar view with a target depth of <4 mm below the aortic annulus [20]. In subsequent years, a cusp-overlap technique aiming for 0–3 mm below the aortic annulus was defined to reduce PPM rates for self-expanding valves. However, in most of our cases, we used a standard co-planar view, targeting a depth of mainly 2–4 mm below the aortic annulus.

### 2.5. Statistical Analysis

Statistical analyses were performed using SPSS for Windows, version 29.0 (SPSS Inc., Chicago, IL, USA). Continuous variables are expressed as means ± standard deviations or medians (Q1–Q3) according to their distribution, and categorical variables are expressed as numbers (percentages). Kaplan–Meier survival analysis was applied to determine survival rates and life expectancy. Multivariate Cox regression analysis was performed to assess the independent predictors of mortality in the overall population (entered variables: age, gender, hypertension, diabetes, chronic obstructive pulmonary disease, previous history of bypass graft, malignancy, STS score, Euroscore, device success, pre-TAVI PCI, pre-TAVI EF, post-TAVI EF, cardiac complications, vascular complications, stroke, PPM requirement, post-TAVI AKI, PVL). A *p*-value of <0.05 was considered to indicate significance.

## 3. Results

### 3.1. Baseline Characteristics

Between June 2012 and December 2023, 375 patients underwent TAVI in our clinic: 361 patients for severe AS, 4 for severe aortic insufficiency, 5 for valve-in-valve, and 5 for valve-in-TAVI. The mean age of the patients was 78.4 ± 7.3 years, and the median STS score was 4.2 (2.9–5.9). The basal mean gradient was 46.9 ± 16.2 mmHg, and the pre-TAVI LVEF was 52.0% ± 13.7% (Table 1).

### 3.2. Procedural Characteristics

**Figure 1:**
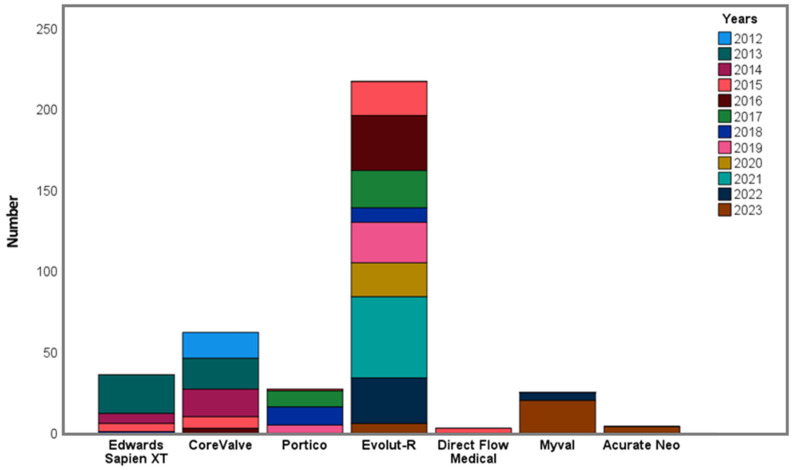
Implanted valves according to year.

**Figure 2:**
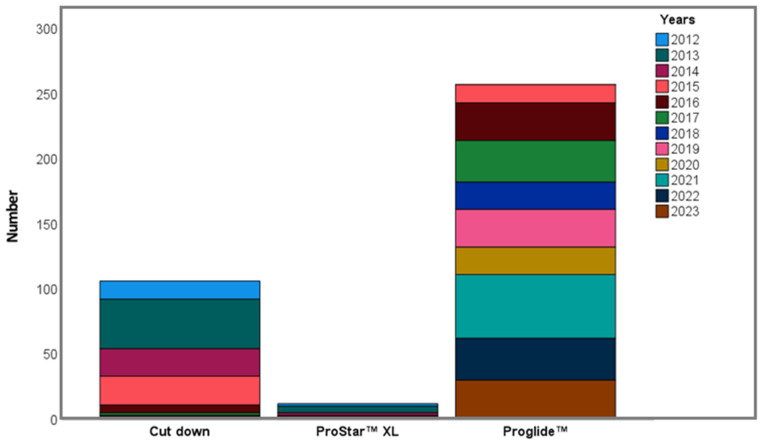
Transfemoral approaches used according to year.

The baseline procedural characteristics of the patients are shown in Table 2. In all, 36 (9.6%) ESVs, 279 (74.6%) Medtronic valves (61 MCVs and 218 Evolut R), 27 (7.2%) Portico valves, 25 (6.7%) Myval, 4 (1.1%) Acurate Neo, and 3 (0.8%) Direct Flow Medical valves were implanted. The valves implanted according to the year are shown in Figure 1. A transfemoral approach was used in all patients. Surgical cutdown was applied in 106 (28.3%) patients, and percutaneous closure systems were applied in 269 (71.7%) patients. The transfemoral approaches according to the year are shown in Figure 2.

### 3.3. Procedural Outcomes

**Figure 3:**
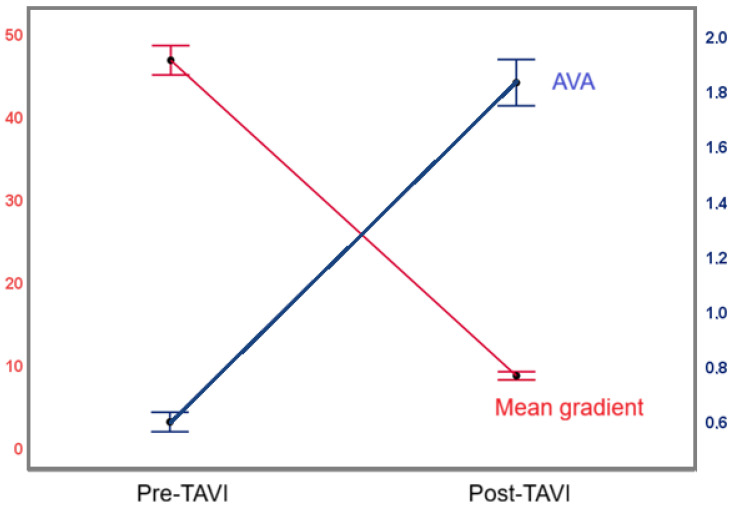
Changes in the mean gradient and aortic valve area following the TAVI procedure.

After TAVI, the mean gradient decreased from 45 (38–55) mm Hg to 8 (5–11) mm Hg, while the AVA increased from 0.6 (0.5–0.7) cm^2^ to 1.8 (1.6–2.0) cm^2^ (Figure 3). The patients’ procedural outcomes are shown in Table 3. According to the VARC-3 criteria, the device success rate was 85.3%, and the technical success rate was 90.4%. Seven (1.9%) patients required a second valve implantation, and one valve-in-valve case could not be performed due to an inability to cross into the left ventricle. The reasons for the second valve implantation were embolization of the prosthetic valve to the ascending aorta in four patients and deep implantation of the prosthetic valve in three patients, resulting in severe AR. Vascular complications were observed in 42 (11.2%) patients; of these, 18 (4.8%) patients experienced major vascular complications. Eleven (61%) cases of major complications were cases in which the percutaneous closure system had been used, and the remaining seven (39%) were cutdown cases. No significant difference was found between percutaneous closure system and cutdown cases (*p* = 0.305). A detailed explanation of the complications and their treatment is presented in Table 4.

### 3.4. Mortality

**Figure 4:**
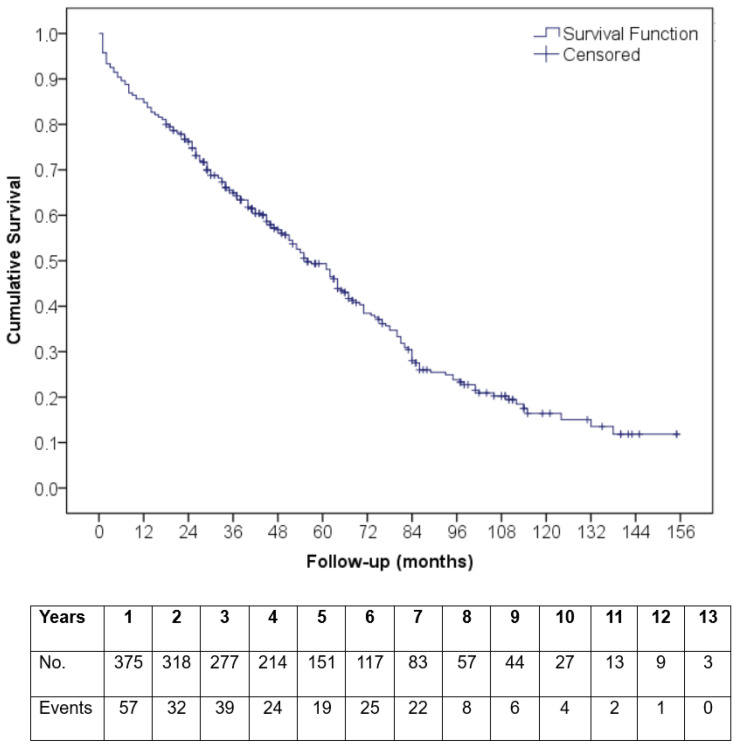
A Kaplan–Meier curve of the overall survival of the total study population.

The median survival time was 56 (49–63) months. A Kaplan–Meier curve of the overall survival of the total study population is presented in Figure 4. The peri-procedural, 30-day, 1-year, and 5-year mortality rates were 5.1%, 4.3%, 15.2%, and 45.6%, respectively. Multivariate Cox regression analysis was performed to determine the independent predictors of overall all-cause mortality. The STS score (HR:1.129, 95% CI: 1.068–1.192, *p* < 0.001) and post-TAVI AKI (HR:3.993, 95% CI: 1.629–9.785, *p* = 0.002) were detected as these independent predictors.

### 3.5. Other Complications

Three (0.8%) patients presented with infective endocarditis (IE) on the 38th, 120th, and 1920th days post-TAVI. The earliest case had staphylococcus aureus on blood culture and underwent surgical explantation of the implanted valve and bioprosthetic valve replacement under antibiotic treatment. The second case had Enterococcus Faecalis in his blood culture and was treated with antibiotics. The latest case had Enterecoccus durans on blood cultures and was treated with antibiotics. She was fine for about 2 years under regular follow-up but needed a valve-in-TAVI for ongoing detrimental valvular severe aortic insufficiency.

Other rare complications comprised ventricular septal defects (VSDs), which were detected in two (0.5%) patients at the first echocardiographic control before discharge, and coronary obstruction, which occurred in one (0.3) patient after implantation of an ESV and was percutaneously treated.

### 3.6. Antiplatelet and/or Anticoagulant Regimen

The antiplatelet and anticoagulant regimens after TAVI are presented in Table 5. The most common frequently used regimen was a combination of aspirin + clopidogrel (59.5%). The next most frequently used treatments were NOACs alone (11.5%) and aspirin or clopidogrel alone (10.9%).

The combination of warfarin + aspirin + clopidogrel was preferred in only 1.6% of patients.

## 4. Discussion

This study examined one of the largest series of TAVI procedures performed in our country, which were conducted with six different types of bioprosthetic valves (mainly four valve types) implanted by the same operators. We presented a reflection of real-world experience of the evolution of TAVI in our registry and demonstrated high procedural success and favorable outcomes in 375 patients.

Over the past decade, patient risk scores in the German registry, as assessed using the STS score, nearly halved from 7.2% to 4.6% [21]. In the French registry, a similar trend in Euroscore 1 was observed over time as compared with the German registry [22]. Conversely, in our registry, the median STS score was 4.2% and remained unchanged over time. This may be because the risk scores of all patients in our registry were re-calculated according to the latest STS and Euroscore II score calculators, as the risk scores used for TAVI patients have changed over time. On the other hand, the 30-day and 1-year mortality rates were 4.2% and 15.2% in our study. The rates in our study are comparable with those in earlier registries, including PARTNER A (3.4% and 24.2%), the FRENCH registry (9.7% and 24.0%), and the PARTNER 2 trial (3.9% and 12.3%). In addition, the findings from our study closely align with those reported by Tuzcu et al., who documented 30-day and 1-year mortality rates of 4.2% and 21.2%, respectively, following native valve TAVI [23]. Notably, their cohort exhibited a median STS score of 6.8%, indicative of intermediate surgical risk [1,23], comparable to the risk profile of our study population. Furthermore, we found that the STS score was an independent predictor of all-cause mortality. Similarly to our study, previous studies also reported STS as an independent predictor of mortality following TAVI [24,25]. Although STS scoring systems are not validated for the prediction of mortality after TAVI, an increased STS score may be considered associated with poor outcomes of TAVI.

Valve design is pivotal in optimizing outcomes across the pre-, intra-, and post-procedural phases. Recent advancements in bioprostheses for TAVI have led to continuous improvements in device performance [26,27]. As a result, technological advances aimed at improving the effectiveness of devices have led to the creation of next-generation bioprostheses, such as the Sapien 3, Sapien 3 Ultra, Sapien 3 Ultra Resilia, Portico Navitor, Evolut R, Evolut PRO, PRO+, Evolut FX, and FX+ systems. These iterations feature an external pericardial skirt to improve sealing and are delivered via smaller-profile inline sheaths, facilitating less invasive implantation [28,29]. In a very recent meta-analysis including 3106 patients who underwent TAVI, the authors showed that Myval’s performance is comparable with that of available transcatheter heart valves regarding safety and effectiveness [30]. With these technological advancements, we increasingly implanted Evolut R and Myval valves. It has been reported that the development of new valve designs has led to a decrease in the incidence of complications [31].

One of the strengths of our registry is demonstrating vascular complications and their treatment in detail. In transfemoral TAVI, the reported rate of major vascular complications ranges from 4.48% to 15.1% [32]. Studies have reported that vascular complication rates have decreased owing to technological advancements in devices and increased operator experience [33]. We performed TAVI via transfemoral access in all patients. In the case of femoral or iliac artery stenosis, we preferred to use the Evolut R because of its lower profile sheath. Vascular complications were observed in 42 (11.2%) patients, of which 18 (41.8%) patients were defined as experiencing major complications according to the VARC-3 definition in the present study. The frequency of major vascular complications in our study was lower than those in the UK TAVI (10.1%), PARTNER 2A (7.9%), and SURTAVI (6%) studies but higher than that in the PARTNER 3 (2.2%) study [33]. Before percutaneous closure systems were adopted, we performed cutdown in nearly the first one-third of the registry. Then, for a short period, we used a relatively complicated Prostar system with which we had higher complication rates, before finally adopting the Proglide system that we still use. Vascular complications need to be diagnosed early and treated immediately. In our study, the treatment of vascular complications was implemented quickly, and the treatments applied were presented in detail. It should be noted that immediate and successful management of vascular complications is of paramount importance in patients undergoing TAVI.

Studies showed that moderate or worse PVL was associated with increased mortality [34]. Its incidence was from 7% to 24% in earlier studies, but this rate has been shown to decrease over time to below 5% with the introduction of newer-generation devices with enhanced sealing mechanisms [35,36]. In our registry, the frequency of moderate or worse PVL was 2.9%. Similarly to our study, a recent study found the frequency of moderate to severe PVL to be 2% after TAVI [34]. The reason for the decrease in the frequency of PVL compared with that in early studies is the lower part of the device being covered with a pericardial skirt in newly developed devices. We also observed this situation in our study and found that the frequency of moderate or worse PVL was lower, which is consistent with the literature. We believe that careful attention to CT for the annulus size, calcification, and its distribution, leading to proper bioprosthetic valve size selection, has also contributed.

The need for PPM following TAVI is a common complication. The reported incidence varies widely, ranging between 5% and 38%, depending on the type of valve used, depth of implantation, patient anatomy, and other clinical factors such as pre-existing conduction disorders [37]. Self-expanding valves are known to be associated with significantly higher PPM rates compared with balloon-expandable valves [38,39]. This is probably because of the greater expansion of self-expanding valves into the left ventricular outflow tract with compression of the septal conduction tissues. The PPM rates of self-expanding valves have been reduced with the implementation of new deployment techniques such as high implantation in a standard co-planar view and, subsequently, the cusp-overlap technique. In our study, 42 (11.2%) patients required PPM implantation after TAVI. In the PARTNER studies, where balloon-expandable valves were used, the rate was between 3.4% and 8.5% [5,40,41]. In the UK TAVI registry, it was slightly below 12%, and in the France TAVI registry, it was around 19% [10,42]. We believe that this result comes from our high implantation strategy explained before, beginning with MCVs and then used with the other self-expanding valves. The fact that previous studies showed a close relationship between an MCV implantation depth of more than 6 mm and conduction tissue injuries also supports our current results [20].

TAVI operators should always be aware of the possibility of cardiac tamponade when hemodynamic collapse occurs unexpectedly during the procedure. The incidence of pericardial tamponade after TAVI varies from 0.9 to 4.3%. It may be caused by right ventricular perforation by a temporary pacemaker electrode, left ventricular perforation by guidewires and catheters that are used for delivering the transcatheter heart valve, or rupture of the aortic annulus during implantation [43]. In our study, pericardial tamponade occurred in eight (2.1%) patients. Emergency pericardiocentesis was performed in all such cases. Pacemaker-induced pericardial tamponade was observed in six patients. Five of these six patients in whom tamponade was related to pacemaker-induced RV perforation were successfully treated. However, it was impossible to treat the tamponade caused by one case of RV and two cases of LV perforation with pericardiocentesis; unfortunately, all three patients died on the same day as the cardiac surgery. We believe that meticulous attention to stable positioning of the LV stiff wires, balloon-tipped pacemaker electrodes, pacing the right ventricular septum rather than the free wall, and pacing with LV guidewire in selected cases will help to reduce the incidence of this complication.

Stroke is one of the most feared complications after TAVI, with reported incidence between 2.3% and 6.7% [44]. Peri-procedural stroke occurs mostly due to the displacement of atherosclerotic debris from the aorta and aortic valve due to manipulation of catheters in the aortic arch, crossing of the calcified valve, balloon dilatation, and valve deployment or peri-procedural AF, whereas stroke after 30 days occurs mainly due to patient-specific factors. These patients have impaired quality of life and poor outcomes [45]. In our registry, ischemic stroke occurred in six (1.6%) patients, all in the first three days, and was treated conservatively. Fortunately, none of the patients had a neurologic disability. The reasons for the lower stroke rate may be the relatively small size of our study and the lack of routine MRI after TAVI, since it is known that the frequency of newly developed silent intracerebral lesions is higher in studies in which MRI is routinely performed [46]. We evaluated all patients through detailed neurological examinations, but MRI was ordered only in the case of neurological symptoms. However, the efforts of our team to not spend an unnecessarily long time in the ascending and arcus aorta with the delivery system and catheters, to avoid carrying thrombus material from descending aorta aneurysms detected on CT before TAVI, to avoid unnecessary balloon pre- and post-dilatation, and to closely monitor anticoagulation during the procedure may have contributed to this result.

Acute kidney injury after TAVI is a known complication and is associated with short- and long-term mortalities [12,47,48,49]. It may be multifactorial, including contrast-induced nephropathy; hypoperfusion during rapid ventricular pacing; and the development of peri-procedural complications, such as bleeding, cardiac, and vascular complications. The reported frequency of AKI after TAVI varies, with rates ranging from 3% to 50% depending on the definition used [48]. In our study, AKI developed in 7.2% of patients following TAVI, which was consistent with the literature. Besides this, we found that AKI was an independent predictor of all-cause mortality in Cox regression analysis. Similarly, to our study, previous studies detected AKI as an independent predictor of short- and long-term mortalities after TAVI. Therefore, we believe that prophylactic measures such as accurately identifying patients at high risk of AKI before TAVI, minimizing contrast agent use, ensuring adequate pre-procedural hydration, and avoiding hypovolemia are vital for all patients.

On the other hand, there is a non-negligible risk of post-procedural bleeding and transfusion for patients receiving TAVI, and bleeding risk remains one of the major determinants of short- and long-term outcomes following TAVI. Jiritano et al. demonstrated that the rate of bleeding events was not different between patients undergoing surgical AVR or TAVI [50]. They also demonstrated that very elderly (age ≥ 80 years) patients submitted to TAVI had higher rates of bleeding events when compared with elderly (age < 80 years) patients, without statistical significance [50]. In our study, 27.4% of cases needed post-operative ES transfusion, but we did not find a correlation between transfusion requirements and adverse outcomes.

Contemporary clinical guidelines endorse lifelong single antiplatelet therapy in post-TAVI patients who are not indicated for anticoagulants, with the aim to decrease ischemic complication rates and reduce bleeding risk [1,51]. However, this was not clearly defined in previous guidelines, and the use of dual antiplatelet therapy for 3–6 months after TAVI has generally been recommended. In addition, treatment regimens could vary from patient to patient depending on the underlying clinical characteristics. In our study, the majority of those discharged with dual antiplatelet therapy were patients who underwent TAVI in 2020 or earlier, or patients who underwent PCI prior to TAVI. This rate will gradually decrease over time because we observed a temporal decline in the DAPT strategy after 2020 in our study, reflecting our clinical adherence to evidence-based recommendations.

Ventricular septal defect is a rare complication of TAVI [52]. Since this registry covered a long period of time, we were able to demonstrate rare complications of TAVI such as VSD. An unusual complication of TAVI, a membranous-type ventricular septal defect (VSD), was detected in two patients (0.5%) at the first echocardiographic control before discharge. The cases were asymptomatic; however, the defects were hemodynamically significant. Both were closed percutaneously in our clinic [53].

### Limitations

The main limitation of our study is its single-center, retrospective design, which may limit the generalizability of our findings. Another limitation is the lack of newer iterations of several TAVI platforms (e.g., Evolut Pro, Sapien 3) in our registry due to institutional reimbursement restrictions. It would also be better to include several additional metrics, such as procedure times and contrast volumes, in this study to validate the issue of a potential learning curve. Finally, in our study, the number of patients who completed over 12 years of follow-up was quite low. This is because some patients were lost during follow-up, and many had not yet reached this long follow-up period. Therefore, this should be considered when interpreting findings regarding long-term survival, especially after 5 years.

## 5. Conclusions

We demonstrated the evolution of TAVI procedures at a single center over 12 years. A high level of collaboration between experienced operators and innovations in devices seem to be the key features for achieving high procedural success and low complication rates.

## Figures and Tables

**Table 1:** Baseline clinical characteristics of the study population.

Variables	All Patients Who Underwent TAVI (n = 375)
Age, years	78.4 ± 7.3
Gender, male (%)	161 (42.9)
HT (%)	314 (83.7)
DM (%)	154 (41.1)
CAD (%)	161 (42.9)
COPD (%)	88 (23.5)
CABG (%)	72 (19.2)
Valve surgery (%)	21 (5.6)
Peripheral artery disease (%)	27 (7.2)
PPM history (%)	19 (5.1)
Malignancy (%)	29 (7.7)
BMI (kg/m^2^)	27.0 ± 4.5
STS score (%)	4.2 (2.9–5.9)
Euroscore II (%)	4.2 (2.7–6.2)
Pre-op mean gradient, mmHg	45 (38–55)
Pre-op AVA, cm^2^	0.6 (0.5–0.7)
Pre-op EF (%)	52.0 ± 13.7

HT: hypertension; DM: diabetes mellitus; CAD: coronary artery disease; COPD: chronic obstructive pulmonary disease; CABG: coronary artery bypass graft; BMI: body mass index; STS: Society of Thoracic Surgeons; AVA: aortic valve area; EF: ejection fraction.

**Table 2:** Baseline procedural characteristics of the study population.

Variables	All Patients Who Underwent TAVI
	(n = 375)
Pre-TAVI PCI (%)	56 (14.9)
Valve type (%)	
ESV	36 (9.6)
CoreValve	61 (16.3)
Evolut R	218 (58.1)
Portico	27 (7.2)
Myval	25 (6.7)
DFM	3 (0.8)
Acurate Neo	4 (1.1)
Aortic valve pre-dilatation (%)	205 (54.7)
Aortic valve post-dilatation (%)	55 (14.7)
Valve-in-TAVI (%)	5 (1.3)
Valve-in-valve (%)	5 (1.3)
Access type	
Surgical cutdown	106 (28.3)
Percutaneous	269 (71.7)
Prostar	12 (4.5)
Proglide	257 (95.5)
Duration of ICU stay, days	3 (3–5)
Anesthesia type (%)	
Endotracheal intubation	109 (29.1)
Local anesthesia	266 (70.9)
Post-operative ES transfusion requirement (%)	103 (27.4)
Number of ES transfusions	1 (0–2)

TAVI: transcatheter aortic valve implantation; PCI: percutaneous coronary intervention; ESV: Edwards SAPIEN valve; DFM: Direct Flow Medical; ICU: intensive care unit, ES: erythrocyte suspension; DAPT: dual antiplatelet therapy.

**Table 3:** Procedure-related complications and outcomes for the study population.

Variables	All Patients Who Underwent TAVI (n = 375)
Successful valve implantation (%)	365 (97.8)
Device success (%)	320 (85.3)
Technical success (%)	339 (90.4)
Device embolization (%)	4 (1.1)
Second valve implantation (%)	7 (1.9)
Cardiac complication (%)	12 (3.2)
Vascular complication (%)	42 (11.2)
Major vascular complication (%)	18 (4.8)
PPM requirement (%)	42 (11.2)
Moderate or worse PVL (%)	11 (2.9)
Stroke (%)	6 (1.6)
Acute kidney injury (%)	27 (7.2)
In-hospital mortality (%)	15 (4.0)

PPM: permanent pacemaker; PVL: paravalvular leak.

**Table 4:** Vascular complications and treatment modalities.

Vascular Complication Type (No. of Cases)	Treatment Modality (No. of Cases)
Significant or total occlusion of common femoral artery (15)	Balloon angioplasty (7)
	Stent implantation (5)
	Surgery (3)
Rupture of common femoral artery (9)	Surgery (8)
	Graft stent implantation (1)
Pseudoaneurysm (7)	Surgery (3)
	Follow-up (4)
AV fistula (3)	Follow-up (1)
	Surgery (2)
Femoral hematoma (3)	Follow-up (1)
	Surgery (2)
Aortic dissection (1)	Follow-up (1)
Deep venous thrombosis (2)	ECHOS—slow thrombolytic injection (1)
	Anticoagulant (1)
Retroperitoneal hematoma (1)	Follow-up (1)
Lower-extremity emboli (1)	Surgery (1)

**Table 5:** Antiplatelet/anticoagulant treatment at time of discharge.

Variables	All Patients Who Underwent TAVI (n = 375)
Aspirin or clopidogrel alone (%)	41 (10.9)
Aspirin + clopidogrel (%)	223 (59.5)
Warfarin alone (%)	25 (6.7)
Warfarin + aspirin (%)	2 (0.5)
Warfarin + clopidogrel (%)	13 (3.5)
Warfarin + aspirin + clopidogrel (%)	6 (1.6)
NOACs alone (%)	43 (11.5)
NOACs + clopidogrel (%)	22 (5.9)

NOACs: new oral anticoagulants.

## Data Availability

The data presented in this study are available upon request from the corresponding author. The data are not publicly available due to the arrangements made by the Ethics Committee.

## Abbreviations

The following abbreviations are used in this manuscript:

TAVI	Transcatheter aortic valve implantation
AS	Aortic stenosis
VARC-3	Valve Academic Research Consortium-3
PPM	Permanent pacemaker implantation
ESV	Edwards SAPIEN valve
MCV	Medtronic CoreValve
DFM	Direct Flow Medical
STS PROM	Society of Thoracic Surgeons Predictive Risk for Mortality
EuroSCORE I, II	Logistic European System for Cardiac Operative Risk Evaluation Scores
PVL	Paravalvular leakage
TTE	Transthoracic echocardiography
LVEF	Left ventricular ejection fraction
AVA	Aortic valve area
TEE	Transesophageal echocardiography
MSCT	Multi-slice computed tomography
MRI	Magnetic resonance imaging
CAG	Coronary angiography
CAD	Coronary artery disease
VSD	Ventricular septal defect
